# Single-dose sufentanil or fentanyl reduces agitation after sevoflurane anesthesia in children undergoing ophthalmology surgery

**DOI:** 10.12669/pjms.305.4483

**Published:** 2014

**Authors:** Peng Liang, Cheng Zhou, Juan Ni, Zhen Luo, Bin Liu

**Affiliations:** 1 Peng Liang, MD, Department of Anesthesiology, West China Hospital, Sichuan University, Chengdu, 610041, China.; 2 Cheng Zhou, PhD, Department of Anesthesiology, West China Hospital, Sichuan University, Chengdu, 610041, China.; 3 Juan Ni, MD, Department of Anesthesiology, West China Second Hospital, Sichuan University, Chengdu, 610041, China.; 4 Zhen Luo, MD, Department of Anesthesiology, West China Hospital, Sichuan University, Chengdu, 610041, China.; 5 Bin Liu, MD, Department of Anesthesiology, West China Hospital, Sichuan University, Chengdu, 610041, China.

**Keywords:** Agitation, Anesthesia, Pediatric, Recovery, Sevoflurane, Volatile anesthetics

## Abstract

***Objectives:*** The trail investigated the effect of small dose sufentanil or fentanyl administrated before the end of surgery in reducing the incidence of emergence agitation after anesthesia with sevoflurane in preschool children undergoing ophthalmology surgery, and the incidence of emergence agitation of sevoflurane anesthesia.

***Methods:*** From September 2011 to January 2012 January, ninety ASA I-II children, aged from 3-7 years, undergoing ophthalmology surgery in West China Hospital, were randomly assigned to three groups to receive intravenous saline, sufentanil 0.1μg/kg or fentanyl 1μg/kg at 20 minutes before the end of the surgery. Children were scored by scoring system for emergence agitation (SSEA), Children’s and Infants’ Postoperative Pain Scale (CHIPPS) score.

***Results:*** The incidence of agitation was 30% in sufentanil group, 36.67% in fentanyl group, and 63.33% in control group. The incidence of sever agitation (SSEA score≥3) was 6.67% in sufentanil group, 23.37% in fentanyl group, and 36.67% in control group. The agitation and pain scores in sufentanil group and fentanyl group were better than those in control group (P<0.05). There was no difference among three groups about time to extubation.

***Conclusions:*** We conclude that the incidence of emergence agitation after sevoflurane anesthesia in children undergoing ophthalmology surgery is up to 63.33%. The single dose of sufentanil or fentanyl can reduce the emergence agitation in children anesthesized with sevoflurane, with no adverse effects. The effect of sufentanil is better than fentanyl.

## INTRODUCTION

Sevoflurane is currently the most popular inhaled anesthetic in pediatric anesthesia. It can take a fast, safe and smooth mask induction and fast recovery for rapid intaking, elimination and an absence of pungent odor. However, sevoflurane has been reported to be associated with emergence agitation in pediatric patients, especially in preschool-aged children.^1^^,^^2^ The reason for postoperative agitation or delirium is unclear. Factors such as pain, premedication drugs, rapid recovery, anxiety, and age have been reported. However, emergence agitation still occurs even if with sufficient analgesia.^3^ A variety of drugs have been used to prophylactically treat the sevoflurane induced agitation, and previously, fentanyl was an effective alternative.^4^^-^^6^ Li x^7^ and Li J^8^ reported sufentanil administrated during induction or before skin incision can decrease the incidence of emergence  of agitation without delaying the recovery time. But the effect of small dose sufentanil administered before the end of surgery has not been proven in previous studies. This double-blinded, placebo-controlled study was designed to evaluate sufentanil administered 20 minutes before the end of surgery to decrease agitation after sevoflurane anesthesia and investigate the incidence of emergence of agitation in a pediatric ophthalmology surgical population, comparing it with the fentanyl and saline

## METHODS

This double-blind and placebo controlled study was approved by the ethics committee of West China Hospital, Sichuan University and written informed consent was obtained from the parents. Ninety children, aged 3–7 years old, undergoing elective inpatient squint correction surgery from September 2011 to January 2012 were included. Anesthesia was induced with 8% sevoflurane in 100% oxygen via a facemask. After injected remifentanyl 2μg/Kg and succinylcholine 1mg/kg, the optimal trachea tube was intubated. Maintenance of anesthesia was with 2-4% sevoflurane in nitrogen/oxygen at 0.5 fraction of inspired oxygen (FiO_2_). Local anesthesia with 1% lidocaine 5 ml was performed by surgeon before incision. Heart rate, noninvasive arterial blood pressure, oxygen saturation and end-tidal concentration of sevoflurane (ETSevo) were recorded before anesthesia induction and every 5 minutes during surgery. Just at about 20 minutes before the end of surgery, all patients were randomly assigned to receive 5 mL saline (control group, n = 30), sufentanil 0.1μg/Kg diluted in 5 mL of saline (sufentanil group, n =30), or fentanyl 1μg/Kg diluted in 5 mL of saline (fentanyl group, n =30) injected. All observers were unaware of the contents of the study drug. At the end of surgery, sevoflurane and nitrous oxide were discontinued simultaneously.

Ventilation was continued at the same settings and a total gas flow of 4 L/min of oxygen. 0.4% oxybuprocaine hydrochloride eye drops was used for postoperative analgesia. After return of sufficient spontaneous ventilation (VT > 8 mL/kg and respiratory rate >12 breaths/min) and the gag reflex, the endotracheal tube was removed and then transferred to the postanesthesia care unit (PACU). The duration of surgery, the time from the end of anesthesia to eye opening (Ta) (defined as the time until eye opening on command) and to extubation were recorded. Before induction we evaluated the basic behavior of separating from parents and cooperation degree for induction for every child using the same evaluating scale with a four-point scale: 1= calm; 2 =not calm but could be easily calmed; 3 =not easily calmed, moderately agitated or restless; and 4=combative, excited, or disoriented.^9^

Children were sent into the PACU and scored by scoring system for emergence agitation (SSEA), modified Aldrete score, the Children’s and Infants’ Postoperative Pain Scale (CHIPPS) score^10^ and visual analogue scales for satification (VASS). SSEA was included 0–4-point scale (0=sleeping; 1=awake, calm; 2=irritable, crying; 3=inconsolable crying; 4=severe restlessness, disorientation, thrashing around). The behaviors of the children were evaluated every 15 minutes during the previous 30 minutes and then every 30 minutes after 30 minutes. The children were transferred to the ward from the PACU when they satisfied the modified Aldrete score≥9. The time taken to achieve these criteria for discharge from the PACU was recorded. In case of agitation in the PACU the first measure was to facilitate parental contact and when this failed midazolam 0.1mg/Kg was administered. Then sufentanil 0.1 μg/Kg was given intravenous if the pain score was ≥5.

For purposes of analysis, grades 0 and 1 in the scale of emergence behavior were considered no agitation and grades 2 to 4 were considered presence of agitation. Grade 3 and 4 was considered sever agitation. Statistical analyses were performed using SPSS for Windows 10.0. Parametric data were compared among the groups using one-way analysis of variance (ANOVA) followed by a Tukey-HSD test. The incidence of side effects was analysed by the χ^2^-test. P < 0.05 was considered as statistically significant.

## RESULTS

Demographic variables including patient age, sex, weight, basic Hb, WBC, ALT, AST, the scores of preoperative behavior were not significantly different among the three groups. (Table-I).

**Table-I:** Demographic data

	***Group S*** ***(n=30)***	***Group F*** ***(n=30)***	***Group C*** ***(n=30)***
Age (Year)	5.12±1.28	4.77±1.31	5.47±1.38
Weight (Kg)	18.55±4.64	17.63±3.58	18.80±3.56
Gender (M/F)	17/13	14/16	13/17
Score of separating from parents	2.00±0.788	2.10±0.712	2.03±0.718
Score of induction with mask	2.13±0.776	2.17±0.648	2.03±0.718

Values given are mean±SD

The data recorded during the surgery (HR, NBP, ETCO_2_, ETSevo) were not significantly different among the three groups. There were no significant differences among three groups(P>0.05) about time of induction, duration of surgery and anesthesia, and time to intubation. Time to awaken the sufentanil group and fentanyl group was longer than the control Group. There was difference between sufentanil group and control group. Length of stay in PACU, in the sufentanil group and fentanyl group were shorter than control group, and there were significant differences in sufentanil group versus control group and fentanyl group versus control group (P<0.05). But the whole duration from the end of surgery to discharge from PACU, there were no differences among three groups(P>0.05). (Table-II)

**Table-II:** Time data of three groups

***Time***	***Group Sufentanil*** ***(n=30)***	***Group Fentanyl*** ***(n=30)***	***Group Control*** ***(n=30)***	***P***
Induction（Sencond）	18.70±6.78	16.47±7.38	15.70±4.68	0.174
Duration of anesthesia（min）	55.33±7.30	60.80±14.31	58.80±14.48	0.548
Duration of surgery（min）	35.83±7.15	38.4±10.71	37.93±10.59	0.239
Time to extubation（min）	8.62±3.66	7.83±4.13	7.13±8.39	0.614
Time to awake（min）	18.53±6.68*	15.07±7.32	11.50±8.23	0.002
Stay in PACU（min）	50.07±10.57*	52.43±10.08＃	63.73±20.34	0.001
Whole duration （min）	70.90±17.83	64.10±12.33	69.82±17.19	0.79

* Group Sufentanil *vs.* Group Control: P<0. 05;

# Group Fentanyl *vs.* Group Control: P<0. 05.Min-minutes

The score of pain in the control group was higher than other two groups (P<0.05), but there was no difference between sufentanil group and fentanyl group(P>0.05). The 5 minutes score of pain after in control group was higher than sufentanil group(P<0.05). There were no differences among three groups in 10 minutes score of pain after emergence. The dosage of sufentanil in PACU, the sufentanil group was the least, the control group was the most, but there were no differences among three groups (P>0.05).

The scores of emergence agitation (EA) were the lowest for the sufentanil group, than for the fentanyl group, and the highest for the control group. There were significant differences in both sufentanil group *vs.* control group and fentanyl group *vs.* control group (P<0. 05), but neither between the sufentanil group and fentanyl group (P>0.05).

For the incidence of emergence agitation (EA%, scores≥2), there was no significant difference between the sufentanil group and fentanyl group (P>0. 05), and there were significant differences in both sufentanil group *vs. *control group and fentanyl group *vs.* control group (P<0. 05). The incidence of severe emergence agitation (SEA%, scores≥3), was statistically significant difference between the sufentanil group and control group (P<0. 05), but there was no statistically significant difference in sufentanil group *vs. *fentanyl group (P=0.073), and fentanyl group *vs.* control group (P>0. 05).

The total amount of the sedative midazolam used (mg) was the lowest for the sufentanil group and the highest for the control group. There was statistically significant difference between the sufentanil group and control group (P<0. 05), and there was no statistically significant difference both in sufentanil group *vs.* fentanyl group and fentanyl group *vs.* control group (P>0. 05). 

The scores of satisfaction evaluation (VASS) made by the anesthesiologist in the PACU were the highest for the sufentanil group and the lowest for the control group. There were statistically significant differences both in sufentanil group *vs.* control group and fentanyl group *vs. *control group (P<0. 05), but there was no statistically significant difference between the sufentanil group and fentanyl group (P>0. 05). (Table-III)

**Table-III:** Comparison of emergence of agitation status of children and the scores of satisfaction evaluation (VASS).

	***Group Sufentanil***	***Group Fentanyl***	***Group Control***	***P***
EA scores	1. 83±1. 18	2. 17±1. 29	2. 87±1. 11	0. 004
EA%	30. 0%*	36. 67%	63. 33%	0. 022
SEA%	6. 67%*△	23. 3%	36. 67%	0. 021
PACU Mida(mg)	0. 500±0. 798*	0. 647±0. 829	1. 003±0. 860	0. 050
Vass scores of anesthetist	7. 87±1. 94*	7. 47±1. 69#	6. 43±1. 76	0. 006
Vass scores of patients’ family	8. 23±1. 83*	7. 67±1. 79#	6. 53±1. 93	0. 002

* Group Sufentanil *vs.* Group Control: P<0. 05;

# Group Fentanyl *vs.* Group Control: P<0. 05;

△ Group Sufentanil *vs .*Group Fentanyl: P<0. 05.

## DISCUSSIONS

The incidence of emergence agitation for children after sevoflurane anesthesia was as high as 10%-67%.^1^^,^^2^ In the study by Sun JH^11^, the incidence of postoperative agitation was significant for the sevoflurane than for the propofol. Besides, Voepel-Lewis^12^ compared the abdominal and perineal operations.The operation on head and neck is an independent risk factor for the emergence agitation in children. This research revealed that the incidence of emergence agitation after sevoflurane anesthesia for the patients of 3-7 years old who had ophthalmic surgery was as high as 63.33%, in which the rate for severe agitation was 36. 67%.

Many factors would affect the behavior of the children after sevoflurane anesthesia. The younger, more emotional and impulsive, less sociable patients who had difficulties in separation from their parents and those with anxious parents had higher incidence of emergence agitation.^13^^,^^14^ We used the Self-rating Scale that provided scores and made statistics on the separation status of the children from their parents and the cooperative conditions in induction when wearing masks. There was no difference for these two scales among the three groups. In this study, all patients had not used any preoperative drugs to avoid the impact of sedative and anticholinergic agent on postoperative behaviors.

Two studies have showed that the recovery time was negatively correlated with the scores of agitation. Shibata^15^ suggested that the residue sevoflurane may be correlated with agitation, or that the sevoflurane internal effect led to this fact. But Cohen^16^ suspected the disassociation status caused by the difference of nerve system recovery after sevoflurane anesthesia made the children sensitive and agitated to the environment. In the study there was no significant difference in the heart rate, mean arterial pressure and ETSevo at each time point among the three groups during operation. There was no patient who showed movement or mild anesthesia. At the end of the surgery sevoflurane was discontinued, we turned up the ventilation with the same standard for 3 minutes. Demirbilek^17^ found some patients would be soothed with parents’ company. In this study, company of a family member was a must during induction and recovery. To avoid the effect of pain on emergence agitation, we selected local anesthesia during surgery and surface anesthesia for postoperative analgesia.

This study concluded that the incidence of emergence agitation after sevoflurane anesthesia in pediatric ophthalmology was very high. After ophthalmologic surgery, the visual distortion, blackness and the resultant sense of fear after conventional eye bandaging could all cause one's emotional changes. It is thus concluded that the ophthalmologic postoperative agitation may be related to the impact of dressing and bandaging on the visual ability as well as the distortion and fear resulted from difficulties in communication with people and contact with the environment.

The previous studies^4^^,^^6^^,^^18^ had revealed the effects of fentanyl on reducing the emergence agitation. On the contrary, Demirbilek^17^ reported fentanyl had no effects on the agitation behavior, but the author neglected the sedative effects of residue thiopental sodium. The incidence of agitation observed was lower than that reported in most literatures and the results were much expected. Our study revealed that a single dose fentanyl or sufentanil contributed to reducing the incidence of emergence agitation. It is well known that sufentanil and fentanyl have the same mechanisms, but compared with fentanyl of equivalent dose, sufentanil had milder effect of respiratory depression with slight sedative effects, so it reduced the risks of the occurrence of postoperative respiratory depression and hyoxemia. The results of the study also showed that sufentanil is better in reducing emergence agitation than fentanyl. We speculate that mechanism may be related to the slight sedative effect of opioid drugs.

In conclusion, this study showed the incidence of emergence agitation after sevoflurane anesthesia for the preschool children of 3-7 years old undergoing ophthalmology surgery was as high as 63.33%, in which that of severe agitation (scores≥3) was 36. 67%. Intravenous injection of 1.0μg/kg of fentanyl or 0.1μg/kg of sufentanil 20 minutes before the end of operation can effectively reduce the incidence of emergence agitation without affecting extubation and recovery. Sufentanil was better than fentanyl of equivalent dose.

## Authors Contribution:

**Peng Liang,** designed and did statistical analysis & writing of manuscript.

**Cheng Zhou, Zhen Luo **did data collection and manuscript editing.

**Juan Ni** did statistical analysis.

**Bin Liu** did review and final approval of manuscript.
